# Supine bicycle exercise blood pressure, heart rate and rate pressure product in patients with normal stress echocardiograms in an unselected chest pain population

**DOI:** 10.1186/s44156-026-00103-9

**Published:** 2026-02-09

**Authors:** J. C. Flynn, B. L. Elliott, A. Macnab, K. Pearce, M. Stout, L. E. Dobson

**Affiliations:** 1 The University of Manchester, Manchester, UK; 2 Manchester University NHS Foundation Trust, Wythenshawe Hospital, Manchester, UK

**Keywords:** Echocardiography, Supine bicycle echocardiography, Treadmill stress echocardiography, Rate pressure product, Exercise capacity

## Abstract

**Background:**

Reference ranges for exercise capacity during supine bicycle stress echocardiography (SBSE) are not well described. The aim of this study was to define supine bicycle exercise parameters in patients with normal stress echocardiograms in an unselected chest pain population.

**Methods:**

260 normal SBSE tests were analysed, recording baseline and peak measurements of heart rate, systolic and diastolic blood pressure, workload in Watts, peak rate pressure product (RPP) and total exercise time during a standardised 25 W ramp protocol.

**Results:**

There were sex-related differences in physiological response to exercise; peak HR was lower in men than women (men 141+/- 11 bpm vs. women 145+/-11 bpm, *p* < 0.01) and peak blood pressure higher in men than women (men 185+/-29mmHg vs. women 169+/-30mmHg, *p* < 0.05). Men exercised for 20.5% longer than women with a 6.8% greater peak rate pressure product (both *p* < 0.05). Age was also associated with differences in peak heart rate (< 40 156 +/- 9 bpm vs. > 70 129+/-6 bpm, *p* < 0.05), peak systolic blood pressure (153+/-27mmHg vs. > 70 188+/-24mmHg, *p* < 0.05) and reduced exercise time across both sexes (< 40 609+/-158 s vs. > 70 475+/- 126 s, *p* < 0.05). Although exercise time reduced with age, higher peak systolic blood pressure measurements meant peak rate pressure product was not different according to age (< 40 23881 +/- 4310 vs. > 70 24343 +/- 3058, *p* = 0.988).

**Conclusion:**

This study established reference values for systolic blood pressure, rate pressure product, workload in Watts and total exercise time during stress echocardiography using a 25 W bicycle ergometer protocol. Despite exercise time reducing with age, peak rate pressure product remains static, suggesting peak rate pressure product may be a better standardised measure of effort than exercise time alone.

## Introduction

 Supine bicycle exercise stress echocardiography (SBSE) is a reliable non-invasive measure of myocardial function, and a normal study has an implied excellent prognosis for individuals with suspected coronary artery disease (CAD) [1].

Treadmill stress echocardiography typically uses the Bruce protocol, and obtains physiological measurements of heart rate (HR) and blood pressure alongside echocardiographic images at 2 stages- baseline and following peak exertion [2]. Normal reference ranges for exercise capacity using treadmill exercise are well-defined [3–6] allowing clinicians to accurately categorise an individual’s exercise performance, which is a powerful predictor of mortality [4].

Compared to the treadmill, the main advantage of SBSE is the ability to obtain images and physiological measurements at various levels of exercise, rather than just following cessation of exercise as performed with the treadmill Bruce protocol [7]. Typically, with SBSE lower HR values will achieve maximal cardiovascular performance when compared to the treadmill, due to a higher systolic blood pressure during SBSE resulting in a higher overall peak rate pressure product (RPP) [8, 9]. Alongside this, SBSE offers a higher sensitivity for detection of myocardial ischaemia compared with treadmill stress echocardiography [10].

There are currently no published data defining the normal reference ranges for exercise capacity using a supine bicycle ergometer for exercise stress echocardiography. The aim of this study is to define reference ranges for systolic blood pressure (SBP), peak RPP, workload in Watts and total exercise time in patients with normal stress echocardiograms in an unselected chest pain population. Previous treadmill-based studies [3, 4] have established reference values using cohorts referred for exercise testing on clinical grounds. Our study adopts a similar approach, drawing on a patient population that mirrors real-world practice, as SBSE is most commonly performed in symptomatic individuals undergoing further investigation. To our knowledge, no comparable studies have been conducted using bicycle stress testing. We therefore aim to establish reference ranges that are both clinically relevant and directly applicable to the population in which SBSE is most frequently employed.

## Methods

### Patient selection

This study initially screened 776 consecutive patients who had undergone SBSE at a large tertiary cardiac centre (Wythenshawe Hospital, Manchester, UK) between the September 2022 and April 2024. All data was recorded systematically in an excel spreadsheet.

**Fig. 1:**
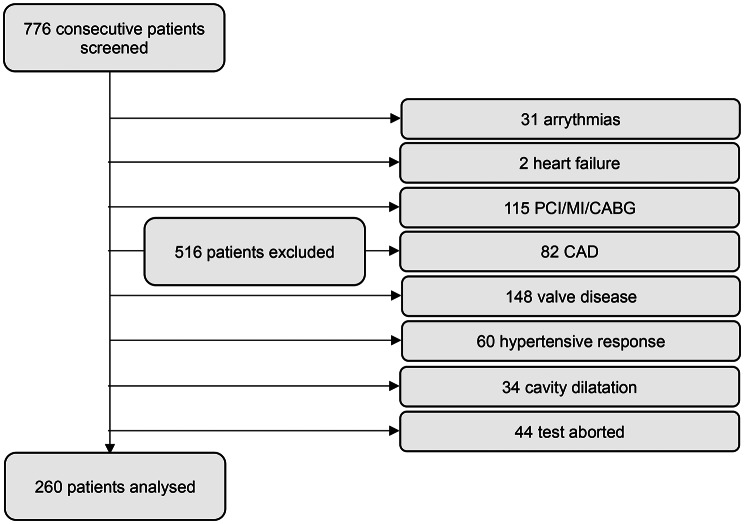
Flow diagram of number of patients included and excluded due to the specified criteria. PCI: percutaneous coronary intervention. MI: myocardial infarction. CABG: coronary artery bypass grafting. CAD: coronary artery disease

Based on this initially recorded data, 516 patients in total were removed via pre-determined exclusion criteria including: a history of arrythmias (31 patients), a history of heart failure (2 patients), a history of CAD, myocardial infarction, previous percutaneous coronary intervention (PCI) or previous coronary artery bypass graft (CABG) (197 patients), ≥moderate valvular heart disease (148 patients), a hypertensive response during the test (60 patients), cavity dilatation during the test (34 patients) or premature test cessation (44 patients). 260 patients were considered adequate for further data collection and retrospective analysis (Fig. 1). A normal stress echocardiogram was defined as normal left ventricular function at rest, > 10% increase in the left ventricular ejection fraction and reduction in left-ventricular cavity size with exercise [11], and absence of inducible regional hypokinesis and symptoms. Data including patient demographics, relevant past medical history (hypertension, diabetes mellitus, history of stroke, history of peripheral arterial disease, history of angina), relevant medications and all stress echocardiography data were obtained. Patients taking beta-blocker medication had it withheld 48 h prior.

### Bike stress echocardiography protocol

SBSE was performed using a supine bicycle and Vivid E Series Cardiovascular Ultrasound system (GE healthcare, Illinois, Chicago). A WHO bike ergometer protocol was used [12] (Table 1) with a constant cadence (between 50 and 60 rpm). Criteria for test termination included the development of symptoms (dyspnoea, leg fatigue or severe chest pain), significant wall motion abnormalities, significant arrythmias or a severe hypertensive response defined as a SBP > 220mmHg and diastolic blood pressure (DBP) > 120mmHg. In the absence of any criteria for test termination, patients were exercised until exhaustion. Peak HR was considered achieved when the participant reached ≥ 85% of their age-predicted maximal HR, calculated as 220 - age.

**Table 1:** Standard bike exercise protocol

Time (mins)	0	2	4	6	8	10	12	14	16
Workload Increments (Watts)	25	50	75	100	125	150	175	190	190

Measurements of blood pressure and HR were recorded at baseline, during the initial 25 W workload, at peak exercise, and during recovery. 3-channel cardiac rhythm monitoring was performed continuously via the echocardiogram machine. Prior to each test, comprehensive baseline images using conventional two-dimensional echocardiography were acquired and evaluated. This included measurements of left ventricle (LV) and right ventricle (RV) size, LV wall motion abnormalities, LV and RV function, aortic root size and aortic valve opening. Assessment for the need for contrast administration was considered if ≥ 2 LV segments could not be visualised. Echocardiographic images obtained at each stage of exercise (baseline, initial 25 W workload, peak exercise, and recovery) included parasternal long-axis, parasternal short-axis (mid-ventricular), and apical 4, apical 2 chamber views.

### Statistical analysis

**Fig. 2:**
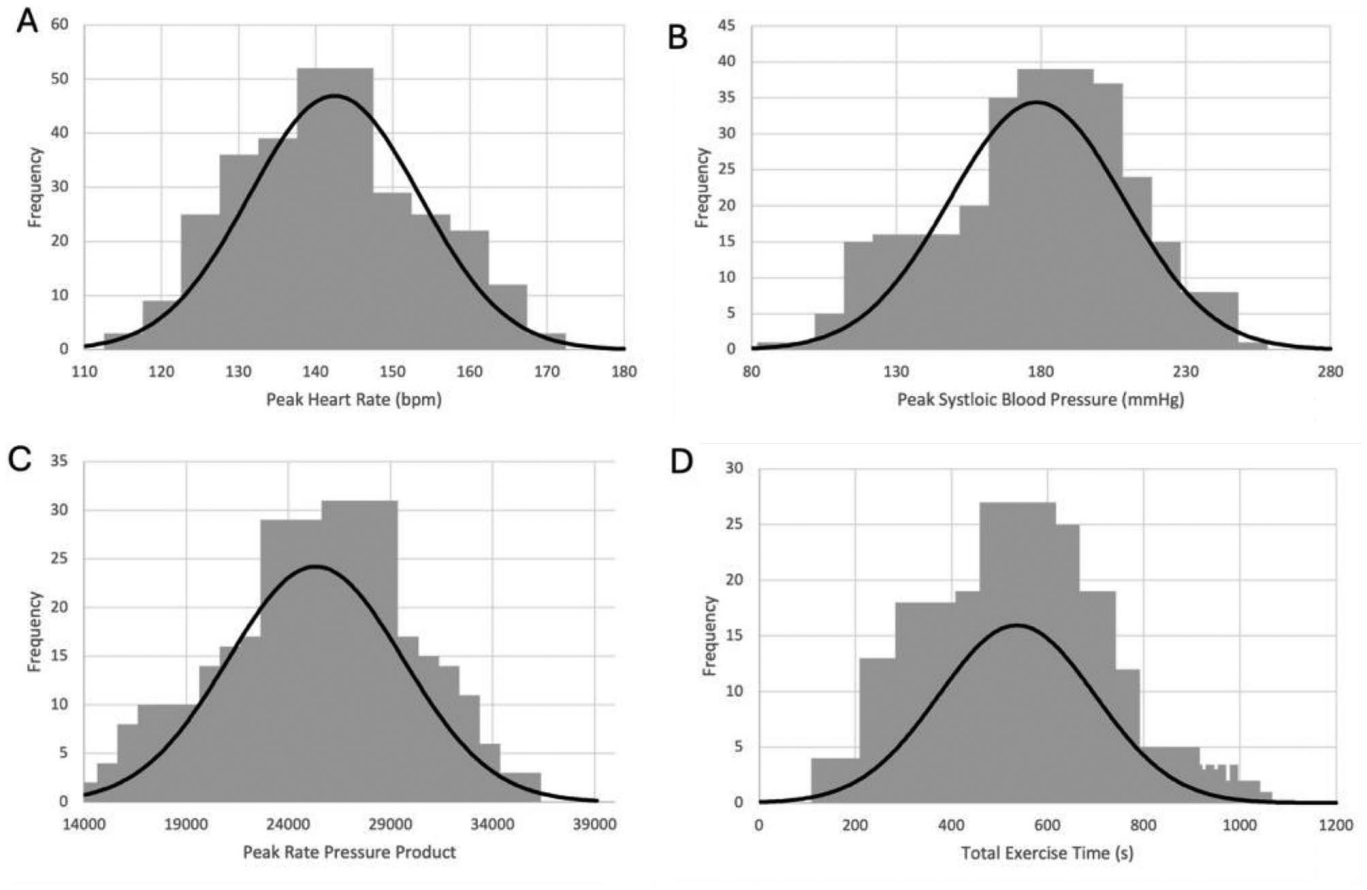
Histograms represent the normal distribution of data for both men and women for peak HR (**A**), peak SBP (**B**), peak RPP (**C**) and total exercise time (**D**)

Based on the SBSE data recorded of 260 patients, RPP was calculated using the standard equation peak HR x peak SBP. Metabolic equivalents (METs) were calculated using the standard equation defined in ACSM’s Guidelines for Exercise Testing and Prescription [13]; METs = [(1.8 x work rate in Watts/body mass in kg) + resting VO2 of 3.5 mL/kg/min + unloaded cycling VO2 of 3.5 mL/kg/min) / 3.5]. Data was then further divided into specified age ranges (< 40, 40–49, 50–59, 60–69 and > 70) and split by gender (male and female). The normal distribution of data was then assessed using histograms (Fig. 2). Using excel graph creator functions, graphs were obtained for each appropriate variables, including SBP, RPP, workload in Watts and total exercise time. Graph data was displayed as mean ± 1.96 standard deviations (SDs) for normally distributed data. Unpaired Student’s T test was performed using excel data analysis tools to evaluate the statistical significance between the gender groups and the < 40 vs. > 70 age categories. ANOVA analysis was used to evaluate the statistical significance between multiple age categories. Tukey’s test was performed as a post-hoc analysis to identify which groups differed significantly.

## Results

Data from 260 patients (153 men and 107 women) were analysed.

### Baseline data

Table 2 shows the mean baseline characteristics according to set age ranges for both men and women combined.

**Table 2:** Baseline characteristics of men and women by age

Age Range	< 40	40–49	50–59	60–69	> 70
Number of Subjects	32	41	67	77	43
Weight, kg	86.2 (20.8)	88 (23.0)	87.9 (18.1)	81.3 (15.3)	78 (16.5)
Height, cm	172.0 (9.3)	171.5 (10.9)	173.4 (9.9)	167.1 (9.3)	168.9 (11.0)
BMI, kg/m^2^	29.2 (7.1)	29.8 (7.1)	29.2 (5.1)	29.2 (5.2)	27.2 (4.5)
Baseline HR, bpm	83 (16)	80 (18)	80 (13)	78 (14)	78 (12)
Resting SBP, mmHg	127(13)	133 (20)	141(19)	145(23)	151(22)
Resting DBP, mmHg	80(11)	80 (16)	83 (10)	79(11)	79 (12)
Hypertension, n (%)	1	13	26	37	27
Diabetes Mellitus, n (%)	3	4	5	11	7
History of Stroke, n (%)	1	0	0	0	0
History of Peripheral Arterial Disease, n (%)	0	0	0	1	0
History of Angina, n (%)	0	0	0	2	1
Medication, n (%)					
ACEi/ARB Beta Blocker CCB Statin Diuretic GTN Aspirin	1 (3) 2 (6) 1 (3) 2 (6) 0 1 (3) 2 (6)	6 (15) 5 (12) 5 (12) 8 (20) 1 (2) 3 (7) 3 (7)	19 (28) 8 (12) 9 (13) 18 (27) 0 9 (13) 6 (9)	24 (31) 5 (6) 27 (35) 37 (48) 6 (8) 26 (34) 21 (27)	13 (30) 7 (16) 18 (42) 23 (53) 3 (7) 16 (37) 13 (30)

Data presented as mean (± SD). HR: heart rate, SBP: systolic blood pressure, DBP: diastolic blood pressure, BMI: Body mass index

Baseline mean heart rate for both sexes was similar across age groups (< 40 83+/-16 bpm vs. > 70 77+/-12 bpm, *p* = 0.397). Baseline mean SBP was increased with advancing age (< 40 127+/-13 bpm vs. > 70 151+/-22 bpm, *p* < 0.05).

### Exercise data

Mean physiological measurements measured at maximal exertion according to set age ranges for both men and women combined is shown in Table 3. With age, mean peak HR was reduced (< 40 156+/-9 bpm vs. > 70 129+/-6 bpm, *p* < 0.05), mean peak SBP was increased (< 40 153+/-27mmHg vs. > 70 188+/-24mmHg, *p* < 0.05) and mean exercise time reduced (< 40 609+/-158 s vs. > 70 475+/- 126 s, *p* < 0.05). Mean peak RPP did not change with age (< 40 23881 +/- 4310 vs. > 70 24343 +/- 3058, *p* = 0.988).

**Table 3:** Physiologic responses at maximal exertion for men and women by age

Age Range	< 40	40–49	50–59	60–69	> 70
Number of Subjects	32	41	67	77	43
Peak HR, bpm	156 (9)	151 (8)	144 (5)	138 (8)	129 (6)
Peak SBP, mmHg	153(27)	173 (29)	182(31)	184(29)	189(24)
Peak DBP, mmHg	87 (17)	89(14)	93 (13)	92 (18)	91 (15)
Peak RPP	23,882(4311)	26,166 (4798)	26,201(4443)	25,277 (4243)	24,343 (3059)
Exercise Time, sec	609(158)	585(183.5)	554(140)	498 (170)	475(126)
Workload, Watts	118(41)	120(45)	110(34)	92 (30)	84(28)
METs	9.6 (1.1)	9.5 (1.1)	9.4 (0.7)	9.0 (0.7)	9.0 (0.7)

Data presented as mean (± SD). HR: heart rate, SBP: systolic blood pressure, DBP: diastolic blood pressure, RPP: rate pressure product

### Baseline and exercise parameters according to sex

The sex-related baseline and exercise characteristics are shown across all age groups are displayed in Tables 4 and 5.

**Table 4:** Baseline and exercise characteristics of men by age

Age Range	< 40	40–49	50–59	60–69	> 70
Number of Subjects	16	22	48	40	27
Weight, kg	90.0 (20.0)	98.6 (20.4)	92.0 (16.7)	87.9 (14.1)	85.0 (13.2)
Height, cm	178.7 (7.0)	179.3 (7.2)	177.8 (7.1)	172.4 (8.4)	174.6 (9.0)
BMI, kg/m^2^	28.1 (5.2)	30.7 (6.0)	29.0 (4.6)	29.8 (5.6)	27.9 (3.8)
Baseline HR, bpm	83.1 (18.7)	76.7 (17.8)	78.7 (13.8)	74.8 (15.6)	75.4 (11.0)
Resting SBP, mmHg	131.0 (9.8)	137.6 (13.4)	142.9 (19.3)	147.6 (18.8)	155.8 (20.1)
Resting DBP, mmHg	85.3 (7.7)	84.3 (11.3)	85.5 (8.7)	82.7 (9.5)	79.4 (12.2)
Peak HR, bpm	155.9 (10.1)	149.6 (7.0)	144.1 (5.2)	136 (7.5)	127.6 (5.4)
Peak SBP, mmHg	167.3 (26.8)	183.5 (27.1)	184.2 (30.1)	187.1 (28.7)	193.6 (26.1)
Peak DBP, mmHg	91.8 (17.4)	90.7 (12.5)	94.4 (12.5)	93.5 (14.7)	91.3 (16.2)
Peak RPP	26042.3 (4309.9)	27465.5 (4310.7)	26543.1 (4387.8)	25442.7 (4176.6)	24674.3 (3265.1)
Exercise Time, s	651.0 (177.8)	629.0 (179.0)	589.0 (142.9)	556.0 (164.4)	498.0 (129.5)
Workload, Watts	135.9 (45.7)	149.8 (38.1)	126.8 (29.2)	106.9 (27.7)	93.7 (28.2)
METs	9.9 (1.3)	9.8 (1.3)	9.5 (0.6)	9.3 (0.7)	9.0 (0.7)

Data represented as mean (± SD). HR: heart rate, SBP: systolic blood pressure, DBP: diastolic blood pressure

**Table 5:** Baseline and exercise characteristics of women by age

Age Range	< 40	40–49	50–59	60–69	> 70
Number of Subjects	16	19	19	37	16
Weigh, kg	82.3 (21.6)	75.8 (19.8)	77.3 (17.4)	74.2 (13.2)	66.2 (15.0)
Height, cm	165.3 (6.0)	162.5 (6.8)	162.2 (6.5)	161.3 (6.1)	159.2 (6.5)
BMI, kg/m^2^	30.4 (8.7)	28.8 (7.7)	29.4 (6.5)	28.5 (4.8)	26.0 (5.5)
Baseline HR, bpm	83.5 (12.6)	84.4 (16.7)	83.6 (8.3)	82.3 (11.7)	81.0 (14.3)
Resting SBP, mmHg	122.8 (13.9)	127.5 (25.2)	136.8 (16.6)	143.1 (26.2)	143.7 (24.4)
Resting DBP, mmHg	74.1 (12.0)	75.8 (19.4)	77.3 (9.7)	75.7 (11.9)	77.0 (10.8)
Peak HR, bpm	156.9 (7.3)	152.3 (9.7)	144.9 (5.0)	140.2 (7.8)	131.9 (5.8)
Peak SBP, mmHg	138.6 (19.5)	161.3 (27.7)	174.9 (31.7)	180.2 (28.7)	180.0 (16.2)
Peak DBP, mmHg	83.1 (15.3)	85.8 (14.7)	90.7 (15.4)	90.0 (20.9)	90.6 (11.5)
Peak RPP	21721.4 (3141.9)	24662.1 (5011.8)	25336.2 (4582.7)	25096.8 (4373.6)	23,785 (2626.8)
Exercise Time, s	567.0 (126.8)	532.0 (179.3)	465.0 (85.7)	433.0 (154.1)	436.0 (113.6)
Workload, Watts	99.7 (26.9)	86.3 (23.5)	81.7 (19.8)	74.3(22.5)	68.8 (19.0)
METs	9.3 (0.9)	9.2 (0.8)	8.9 (0.6)	8.7 (0.7)	8.9 (0.7)

Data presented as mean (± SD). HR: heart rate, SBP: systolic blood pressure, DBP: diastolic blood pressure

The mean baseline HR in men was lower than women (men 77 +/- 15 bpm vs. women 83+/-13 bpm, *p* < 0.05). Mean baseline SBP in men was 6.1% higher than women (men 143 +/- 19mmHg vs. women 135 +/- 24mmHg, *p* < 0.05).

The mean peak HR was lower in men than women (men 141+/- 11 bpm vs. women 145+/-11 bpm, *p* < 0.01). Change in mean heart rate was similar across both sexes (men 64+/-17 bpm, women 62+/- 16, *p* = 0.29). Mean peak SBP was 9.7% higher in men than women (men 185+/-29mmHg vs. women 169+/-30mmHg, *p* < 0.05). Change in mean SBP was similar between sexes (men 40+/- 34mmHg vs. women 34+/-33mmHg, *p* = 0.12). The mean total exercise time in men was 20.5% longer than women (men 577+/-161 s vs. women 477+/-147 s, *p* < 0.05). Mean peak RPP was greater in men than women (men 26000+/- 4175 vs. 24348+/-4276, *p* < 0.05).

**Fig. 3:**
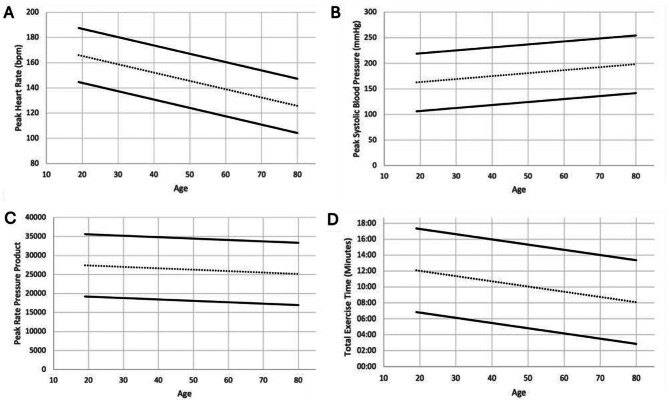
Nomogram displaying peak HR (**A**), peak SBP (**B**), peak RPP (**C**) and exercise time (**D**) for men. The centre dashed line represents the mean. The upper and lower solid lines represent the mean ± 1.96 standard deviations

**Fig. 4:**
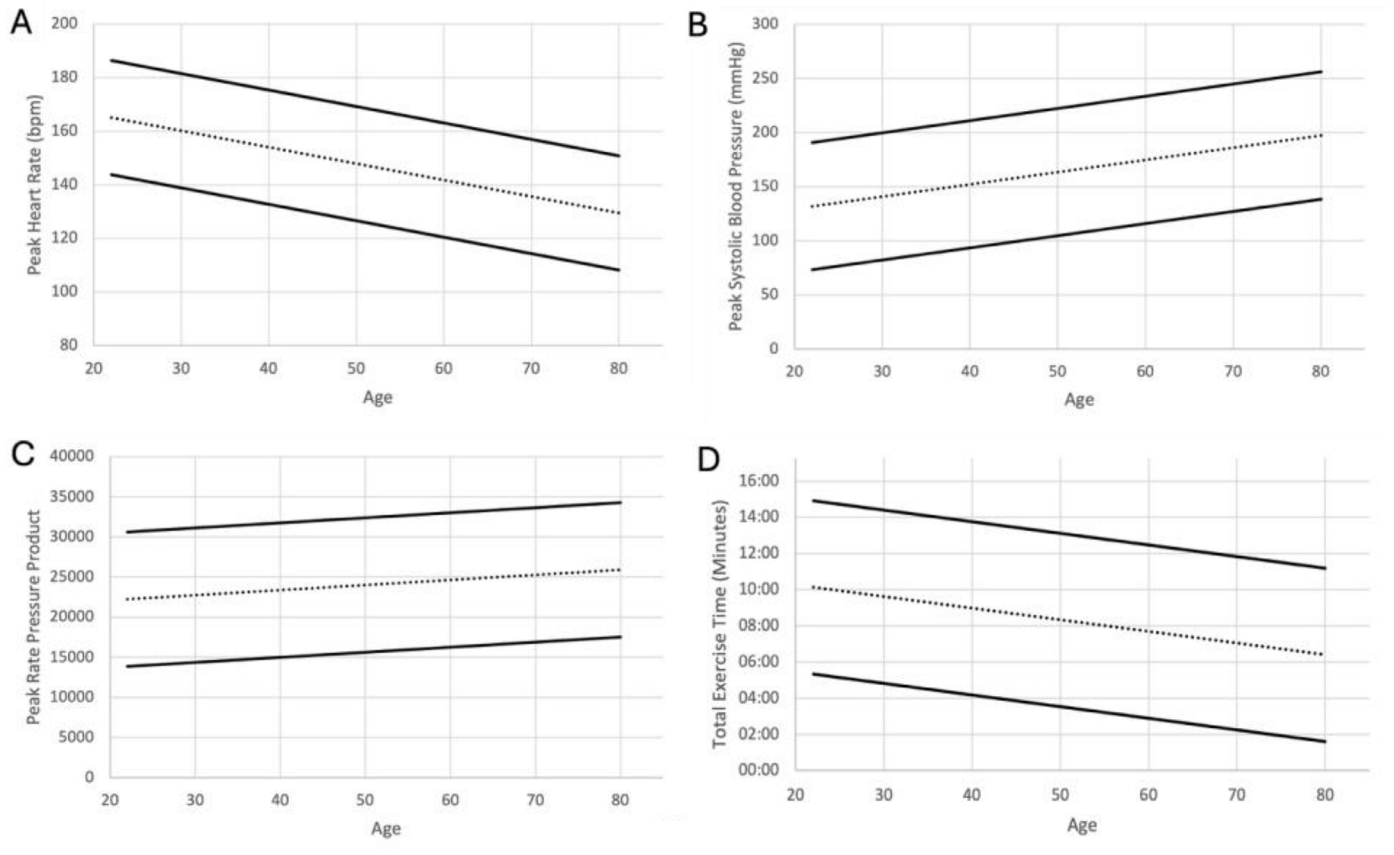
Nomogram displaying peak HR (**A**), peak SBP (**B**), peak RPP (**C**) and exercise time (**D**) for women. The centre dashed line represents the mean. The upper and lower solid lines represent the mean ± 1.96 standard deviations

Nomograms demonstrating average exercise parameters for men and women can be seen in Figs. 3 and 4.

### Statistical analysis comparing age categories for men and women combined

Table 6 shows ANOVA analysis of the comparison of baseline and exercise data across age categories for both men and women combined (< 40, 40–49, 50–59, 60–69, and > 70 years). Tables 7, 8, 9, 10, 11, 12, 13, 14 and 15 show Tukey’s test post-hoc analysis across age categories for men and women combined.

**Table 6:** Single factor ANOVA comparing age categories for men and women combined

Variable	Age groups compared	df	F-statistic	*p*-value
Weight, kg	< 40, 40–49, 50–59, 60–69, > 70	4	2.93	< 0.05
Height, cm	< 40, 40–49, 50–59, 60–69, > 70	4	25.88	< 0.05
BMI, kg/m^2^	< 40, 40–49, 50–59, 60–69, > 70	4	1.35	0.25
Baseline HR, bpm	< 40, 40–49, 50–59, 60–69, > 70	4	0.97	0.43
Resting SBP, mmHg	< 40, 40–49, 50–59, 60–69, > 70	4	9.23	< 0.05
Peak HR, bpm	< 40, 40–49, 50–59, 60–69, > 70	4	89.8	< 0.05
Peak SBP, mmHg	< 40, 40–49, 50–59, 60–69, > 70	4	9.04	< 0.05
Peak RPP	< 40, 40–49, 50–59, 60–69, > 70	4	2.56	< 0.05
Exercise Time, s	< 40, 40–49, 50–59, 60–69, > 70	4	5.71	< 0.05

**Table 7:** Tukey’s test post-hoc analysis for weight categories for men and women combined

Comparison	Mean	95% CI	*p*-value
< 40 vs. 40–49	1.89	-10.0-13.7	0.992
< 40 vs. 50–59	1.70	-9.1-12.5	0.993
< 40 vs. 60–69	4.82	-5.7-15.4	0.720
< 40 vs. > 70	8.17	-3.6-19.9	0.313
40–49 vs. 50–59	0.19	-9.8-10.1	1.000
40–49 vs. 60–69	6.71	-3.0-16.4	0.321
40–49 vs. > 70	10.06	-0.9-21.0	0.089
50–59 vs. 60–69	6.52	-1.9-14.9	0.208
50–59 vs. > 70	9.87	0.1–19.7	< 0.05
60–69 vs. > 70	3.35	-6.2-12.9	0.872

**Table 8:** Tukey’s test post-hoc analysis for height categories for men and women combined

Comparison	Mean	95% CI	*p*-value
< 40 vs. 40–49	19.83	-11.8-51.4	0.423
< 40 vs. 50–59	79.39	47.8–111.0	< 0.05
< 40 vs. 60–69	95.60	64.0-127.2	< 0.05
< 40 vs. > 70	22.82	-8.8-54.4	0.278
40–49 vs. 50–59	59.56	27.9–91.2	< 0.05
40–49 vs. 60–69	75.77	44.2-107.4	< 0.05
40–49 vs. > 70	2.99	-28.6-34.6	0.999
50–59 vs. 60–69	16.21	-15.4-47.8	0.625
50–59 vs. > 70	56.57	25.0-88.2	< 0.05
60–69 vs. > 70	72.78	41.2-104.4	< 0.05

**Table 9:** Tukey’s test post-hoc analysis for BMI categories for men and women combined

Comparison	Mean	95% CI	*p*-value
< 40 vs. 40–49	0.57	-3.1-4.2	0.993
< 40 vs. 50–59	0.08	-3.2-3.4	1.000
< 40 vs. 60–69	0.06	-3.2-3.3	1.000
< 40 vs. > 70	2.04	-1.6-5.7	0.532
40–49 vs. 50–59	0.65	-2.4-3.7	0.977
40–49 vs. 60–69	0.63	-2.4-3.6	0.978
40–49 vs. > 70	2.61	-0.8-6.0	0.215
50–59 vs. 60–69	0.02	-2.6-2.6	1.000
50–59 vs. > 70	1.96	-1.1-5.0	0.389
60–69 vs. > 70	1.97	-1.0-4.9	0.353

**Table 10:** Tukey’s test post-hoc analysis for baseline HR categories for men and women combined

Comparison	Mean	95% CI	*p*-value
< 40 vs. 40–49	3.14	-6.2-12.5	0.888
< 40 vs. 50–59	3.22	-5.2-11.7	0.833
< 40 vs. 60–69	4.96	-3.3-13.3	0.472
< 40 vs. > 70	5.93	-3.3-15.2	0.397
40–49 vs. 50–59	0.09	-7.8-8.0	1.000
40–49 vs. 60–69	1.82	-5.9-9.5	0.966
40–49 vs. > 70	2.79	-5.9-11.5	0.903
50–59 vs. 60–69	1.73	-4.9-8.3	0.951
50–59 vs. > 70	2.71	-5.0-10.5	0.872
60–69 vs. > 70	0.97	-6.6-8.5	0.997

**Table 11:** Tukey’s test post-hoc analysis for resting SBP categories for men and women combined

Comparison	Mean	95% CI	*p*-value
< 40 vs. 40–49	6.17	-6.9-19.3	0.696
< 40 vs. 50–59	14.29	2.4–26.2	< 0.05
< 40 vs. 60–69	18.51	6.9–30.2	< 0.05
< 40 vs. > 70	24.57	11.6–37.5	< 0.05
40–49 vs. 50–59	8.12	-2.9-19.2	0.260
40–49 vs. 60–69	12.35	1.5–23.1	< 0.05
40–49 vs. > 70	18.40	6.2–30.6	< 0.05
50–59 vs. 60–69	4.23	-5.0-13.5	0.720
50–59 vs. > 70	10.28	-0.6-21.2	0.074
60–69 vs. > 70	6.06	-4.6-16.7	0.522

**Table 12:** Tukey’s test post-hoc analysis for peak HR categories for men and women combined

Comparison	Mean	95% CI	*p*-value
< 40 vs. 40–49	5.50	0.8–10.2	< 0.05
< 40 vs. 50–59	12.02	7.8–16.2	< 0.05
< 40 vs. 60–69	18.46	14.3–22.6	< 0.05
< 40 vs. > 70	27.19	22.6–31.8	< 0.05
40–49 vs. 50–59	6.51	2.6–10.5	< 0.05
40–49 vs. 60–69	12.95	9.1–16.8	< 0.05
40–49 vs. > 70	21.69	17.3–26.0	< 0.05
50–59 vs. 60–69	6.44	3.1–9.7	< 0.05
50–59 vs. > 70	15.17	11.3–19.0	< 0.05
60–69 vs. > 70	8.73	5.0-12.5	< 0.05

**Table 13:** Tukey’s test post-hoc analysis for peak SBP categories for men and women combined

Comparison	Mean	95% CI	*p*-value
< 40 vs. 40–49	20.32	1.7–38.9	0.025
< 40 vs. 50–59	28.63	11.8–45.4	< 0.05
< 40 vs. 60–69	30.89	14.4–47.4	< 0.05
< 40 vs. > 70	36.04	17.6–54.5	< 0.05
40–49 vs. 50–59	8.31	-7.4-24.0	0.595
40–49 vs. 60–69	10.57	-4.9-26.0	0.331
40–49 vs. > 70	15.72	-1.7-33.2	0.100
50–59 vs. 60–69	2.26	-10.9-15.4	0.990
50–59 vs. > 70	7.41	-8.1-22.9	0.682
60–69 vs. > 70	5.15	-10.1-20.4	0.885

**Table 14:** Tukey’s test post-hoc analysis for peak RPP categories for men and women combined

Comparison	Mean	95% CI	*p*-value
< 40 vs. 40–49	2289.78	-484.1-5063.7	0.159
< 40 vs. 50–59	2319.02	-180.0-4818.1	0.083
< 40 vs. 60–69	1399.78	-1065.9-3865/4	0.525
< 40 vs. > 70	488.80	-2254.4-3232.0	0.988
40–49 vs. 50–59	29.25	-2313.1-2371.6	1.000
40–49 vs. 60–69	890.00	-1416.7-3196.7	0.827
40–49 vs. > 70	1800.98	-800.3-4402.3	0.319
50–59 vs. 60–69	919.25	-1048.3-2886.8	0.701
50–59 vs. > 70	1830.23	-475.7-4136.2	0.190
60–69 vs. > 70	910.98	-1358.7-3180.7	0.805

**Table 15:** Tukey’s test post-hoc analysis for exercise time categories for men and women combined

Comparison	Mean	95% CI	*p*-value
< 40 vs. 40–49	24.82	-76.9-126.6	0.963
< 40 vs. 50–59	55.27	-37.4-148.0	0.475
< 40 vs. 60–69	111.77	21.0-202.5	< 0.05
< 40 vs. > 70	134.08	33.4-234.8	< 0.05
40–49 vs. 50–59	30.45	-55.1-116.0	0.865
40–49 vs. 60–69	86.95	3.5-170.4	< 0.05
40–49 vs. > 70	109.26	15.1-203.4	< 0.05
50–59 vs. 60–69	56.50	-15.6-128.6	0.201
50–59 vs. > 70	78.81	-5.5-163.1	0.079
60–69 vs. > 70	22.31	-59.8-104.4	0.945

ANOVA revealed a statistically significant difference between age groups for weight (*p* < 0.05), height (*p* < 0.05), resting SBP (*p* < 0.05), peak HR (*p* < 0.05), peak SBP (*p* < 0.05), peak RPP (*p* < 0.05), and exercise time (*p* < 0.05). BMI and baseline HR did not differ significantly between age categories (*p* = 0.25); *p* = 0.43).

Post-hoc Tukey’s analysis demonstrated that the significant differences were predominantly observed between the younger and older age groups. Specifically, weight differed significantly between those aged 50–59 and > 70 years (*p* < 0.05). For height, multiple pairwise differences were observed: <40 vs. 50–59, < 40 vs. 60–69, 40–49 vs. 50–59, 40–49 vs. 60–69, 50–59 vs. > 70, and 60–69 vs. > 70 (*p* < 0.05). Resting SBP was significantly higher in the older age groups, with differences seen between < 40 vs. 50–59, < 40 vs. 60–69, < 40 vs. > 70, 40–49 vs. 60–69, and 40–49 vs. > 70 (*p* < 0.05).

Peak HR showed a significant decline with increasing age, with all pairwise comparisons reaching significance (*p* < 0.05). Peak SBP was significantly higher in the older age groups, with differences between < 40 vs. 50–59, < 40 vs. 60–69, and < 40 vs. > 70 (*p* < 0.05). Although peak RPP showed significance on ANOVA, no individual Tukey’s pairwise comparison reached statistical significance (*p* > 0.05). Exercise time significantly declined with advancing age, with differences between < 40 vs. 60–69, < 40 vs. > 70, 40–49 vs. 60–69, and 40–49 vs. > 70 (*p* < 0.05).

### Statistical analysis comparing age categories for men

Table 16 shows ANOVA analysis of baseline and exercise data across age categories in men. Tables 17, 18, 19, 20, 21, 22, 23, 24, 25, 26, 27, 28, 29, 30, 31, 32, 33 and 34 show Tukey’s test post-hoc analysis across age categories for men and women separately.

**Table 16:** Single factor ANOVA comparing age categories for men

Variable	Age groups compared	df	F-statistic	*p*-value
Weight, kg	< 40, 40–49, 50–59, 60–69, > 70	4	1.93	0.110
Height, cm	< 40, 40–49, 50–59, 60–69, > 70	4	10.12	< 0.05
BMI, kg/m^2^	< 40, 40–49, 50–59, 60–69, > 70	4	1.02	0.399
Baseline HR, bpm	< 40, 40–49, 50–59, 60–69, > 70	4	0.19	0.944
Resting SBP, mmHg	< 40, 40–49, 50–59, 60–69, > 70	4	3.28	< 0.05
Peak HR, bpm	< 40, 40–49, 50–59, 60–69, > 70	4	30.4	< 0.05
Peak SBP, mmHg	< 40, 40–49, 50–59, 60–69, > 70	4	8.04	< 0.05
Peak RPP	< 40, 40–49, 50–59, 60–69, > 70	4	2.21	0.07
Exercise Time, s	< 40, 40–49, 50–59, 60–69, > 70	4	3.67	< 0.05

**Table 17:** Tukey’s test post-hoc analysis for weight categories for men

Comparison	Mean	95% CI	*p*-value
< 40 vs. 40–49	6.49	-9.5-22.4	0.790
< 40 vs. 50–59	4.96	-11.0-20.9	0.909
< 40 vs. 60–69	8.08	-6.0-22.1	0.504
< 40 vs. > 70	16.05	-0.6-32.7	0.063
40–49 vs. 50–59	1.52	-13.7-16.8	0.999
40–49 vs. 60–69	1.59	-11.7-14.9	0.997
40–49 vs. > 70	9.56	-6.4-25.5	0.459
50–59 vs. 60–69	3.11	-10.2-16.4	0.966
50–59 vs. > 70	11.09	-4.9-27.0	0.308
60–69 vs. > 70	7.97	-6.1-22.0	0.517

**Table 18:** Tukey’s test post-hoc analysis for weight categories for women

Comparison	Mean	95% CI	*p*-value
< 40 vs. 40–49	8.59	-6.3-23.5	0.507
< 40 vs. 50–59	2.00	-11.1-15.1	0.993
< 40 vs. 60–69	2.10	-11.3-15.5	0.993
< 40 vs. > 70	5.07	-9.3-19.4	0.865
40–49 vs. 50–59	6.59	-5.1-18.3	0.528
40–49 vs. 60–69	10.69	-1.4-22.8	0.109
40–49 vs. > 70	13.67	0.6–26.7	0.035
50–59 vs. 60–69	4.10	-5.6-13.8	0.772
50–59 vs. > 70	7.07	-3.9-18.0	0.385
60–69 vs. > 70	2.98	-8.3-14.3	0.950

**Table 19:** Tukey’s test post-hoc analysis for height categories for men

Comparison	Mean	95% CI	*p*-value
< 40 vs. 40–49	11.95	-35.1-59.0	0.956
< 40 vs. 50–59	11.78	-35.2-58.8	0.958
< 40 vs. 60–69	89.81	42.8-136.8	< 0.05
< 40 vs. > 70	2.65	-44.4-49.7	1.000
40–49 vs. 50–59	0.16	-46.9-47.2	1.000
40–49 vs. 60–69	77.86	30.8-124.9	< 0.05
40–49 vs. > 70	14.59	-32.4-61.6	0.913
50–59 vs. 60–69	78.03	31.0-125.0	< 0.05
50–59 vs. > 70	14.43	-32.6-61.4	0.916
60–69 vs. > 70	92.46	45.4-139.5	< 0.05

**Table 20:** Tukey’s test post-hoc analysis for height categories for women

Comparison	Mean	95% CI	*p*-value
< 40 vs. 40–49	22.60	-19.0-64.2	0.567
< 40 vs. 50–59	118.27	76.7-159.9	< 0.05
< 40 vs. 60–69	84.13	42.5-125.7	< 0.05
< 40 vs. > 70	38.65	-2.9-80.2	0.082
40–49 vs. 50–59	95.67	54.1-137.3	< 0.05
40–49 vs. 60–69	61.52	19.9-103.1	< 0.05
40–49 vs. > 70	16.04	-25.5-57.6	0.827
50–59 vs. 60–69	34.15	-7.4-75.7	0.163
50–59 vs. > 70	79.63	38.0-121.2	< 0.05
60–69 vs. > 70	45.48	3.9–87.1	< 0.05

**Table 21:** Tukey’s test post-hoc analysis for BMI categories for men

Comparison	Mean	95% CI	*p*-value
< 40 vs. 40–49	1.56	-4.5-7.6	0.953
< 40 vs. 50–59	0.95	-5.1-7.0	0.992
< 40 vs. 60–69	1.87	-3.5-7.2	0.866
< 40 vs. > 70	4.33	-2.0-10.6	0.320
40–49 vs. 50–59	0.61	-5.2-6.4	0.998
40–49 vs. 60–69	0.31	-4.7-5.4	1.000
40–49 vs. > 70	2.77	-3.3-8.8	0.709
50–59 vs. 60–69	0.92	-4.1-6.0	0.986
50–59 vs. > 70	3.38	-2.7-9.4	0.533
60–69 vs. > 70	2.46	-2.9-7.8	0.705

**Table 22:** Tukey’s test post-hoc analysis for BMI categories for women

Comparison	Mean	95% CI	*p*-value
< 40 vs. 40–49	2.57	-2.0-7.1	0.528
< 40 vs. 50–59	0.96	-3.0-5.0	0.964
< 40 vs. 60–69	1.70	-2.4-5.8	0.783
< 40 vs. > 70	0.21	-4.2-4.6	1.000
40–49 vs. 50–59	1.61	-2.0-5.2	0.725
40–49 vs. 60–69	0.87	-2.8-4.6	0.966
40–49 vs. > 70	2.78	-1.2-6.8	0.309
50–59 vs. 60–69	0.74	-2.2-3.7	0.959
50–59 vs. > 70	1.17	-2.2-4.5	0.870
60–69 vs. > 70	1.91	-1.6-5.4	0.549

**Table 23:** Tukey’s test post-hoc analysis for baseline HR categories for men

Comparison	Mean	95% CI	*p*-value
< 40 vs. 40–49	0.89	-11.3-13.0	1.000
< 40 vs. 50–59	0.08	-11.9-12.1	1.000
< 40 vs. 60–69	1.22	-9.4-11.8	0.998
< 40 vs. > 70	2.50	-10.2-15.2	0.982
40–49 vs. 50–59	0.81	-10.8-12.4	1.000
40–49 vs. 60–69	2.11	-8.1-12.3	0.978
40–49 vs. > 70	3.39	-9.0-15.8	0.941
50–59 vs. 60–69	1.30	-8.7-11.3	0.996
50–59 vs. > 70	2.58	-9.6-14.8	0.977
60–69 vs. > 70	1.28	-9.6-12.1	0.997

**Table 24:** Tukey’s test post-hoc analysis for baseline HR categories for women

Comparison	Mean	95% CI	*p*-value
< 40 vs. 40–49	6.40	-7.2-20.0	0.694
< 40 vs. 50–59	4.42	-7.6-16.4	0.847
< 40 vs. 60–69	8.30	-4.0-20.6	0.340
< 40 vs. > 70	7.75	-5.3-20.9	0.477
40–49 vs. 50–59	1.98	-8.7-12.7	0.986
40–49 vs. 60–69	1.90	-9.1-12.9	0.989
40–49 vs. > 70	1.36	-10.6-13.3	0.998
50–59 vs. 60–69	3.88	-5.0-12.8	0.747
50–59 vs. > 70	3.34	-6.6-13.3	0.888
60–69 vs. > 70	0.55	-9.8-10.9	1.000

**Table 25:** Tukey’s test post-hoc analysis for resting SBP categories for men

Comparison	Mean	95% CI	*p*-value
< 40 vs. 40–49	4.69	-17.0-26.4	0.975
< 40 vs. 50–59	14.03	-7.4-35.4	0.368
< 40 vs. 60–69	20.24	1.3–39.2	< 0.05
< 40 vs. > 70	20.85	-1.8-43.5	0.087
40–49 vs. 50–59	9.34	-11.4-30.1	0.722
40–49 vs. 60–69	15.56	-2.7-33.8	0.132
40–49 vs. > 70	16.17	-5.9-38.2	0.257
50–59 vs. 60–69	6.21	-11.7-24.1	0.870
50–59 vs. > 70	6.82	-15.0-28.6	0.907
60–69 vs. > 70	0.61	-18.8-20.0	1.000

**Table 26:** Tukey’s test post-hoc analysis for resting SBP categories for women

Comparison	Mean	95% CI	*p*-value
< 40 vs. 40–49	6.64	-9.6-22.8	0.790
< 40 vs. 50–59	11.92	-2.3-26.1	0.147
< 40 vs. 60–69	16.55	2.0-31.1	< 0.05
< 40 vs. > 70	24.81	9.3–40.4	< 0.05
40–49 vs. 50–59	5.28	-7.4-18.0	0.780
40–49 vs. 60–69	9.91	-3.2-23.0	0.229
40–49 vs. > 70	18.18	4.0-32.3	< 0.05
50–59 vs. 60–69	4.63	-5.9-15.2	0.744
50–59 vs. > 70	12.90	1.0-24.8	< 0.05
60–69 vs. > 70	8.26	-4.0-20.5	0.344

**Table 27:** Tukey’s test post-hoc analysis for peak HR categories for men

Comparison	Mean	95% CI	*p*-value
< 40 vs. 40–49	4.54	-2.5-11.6	0.390
< 40 vs. 50–59	11.93	4.9–18.9	< 0.05
< 40 vs. 60–69	16.64	10.4–22.9	< 0.05
< 40 vs. > 70	24.94	17.6–32.2	< 0.05
40–49 vs. 50–59	7.39	0.6–14.2	< 0.05
40–49 vs. 60–69	12.10	6.1–18.1	< 0.05
40–49 vs. > 70	20.40	13.3–27.5	< 0.05
50–59 vs. 60–69	4.71	-1.2-10.6	0.182
50–59 vs. > 70	13.01	6.0–20.0	< 0.05
60–69 vs. > 70	8.30	2.0-14.5	< 0.05

**Table 28:** Tukey’s test post-hoc analysis for peak HR categories for women

Comparison	Mean	95% CI	*p*-value
< 40 vs. 40–49	6.26	0.1–12.5	< 0.05
< 40 vs. 50–59	11.75	6.4–17.1	< 0.05
< 40 vs. 60–69	19.93	14.4–25.5	< 0.05
< 40 vs. > 70	28.32	22.4–34.2	< 0.05
40–49 vs. 50–59	5.49	0.6–10.4	< 0.05
40–49 vs. 60–69	13.67	8.6–18.7	< 0.05
40–49 vs. > 70	22.06	16.6–27.5	< 0.05
50–59 vs. 60–69	8.18	4.2–12.2	< 0.05
50–59 vs. > 70	16.57	12.1–21.1	< 0.05
60–69 vs. > 70	8.39	3.7–13.0	< 0.05

**Table 29:** Tukey’s test post-hoc analysis for peak SBP categories for men

Comparison	Mean	95% CI	*p*-value
< 40 vs. 40–49	22.77	-2.6-48.1	0.099
< 40 vs. 50–59	36.33	11.3–61.3	< 0.05
< 40 vs. 60–69	41.64	19.4–63.9	< 0.05
< 40 vs. > 70	41.44	14.5–68.4	< 0.05
40–49 vs. 50–59	13.56	-10.7-37.8	0.530
40–49 vs. 60–69	18.87	-2.5-40.2	0.110
40–49 vs. > 70	18.67	-7.6-44.9	0.286
50–59 vs. 60–69	5.31	-15.7-26.3	0.956
50–59 vs. > 70	5.11	-20.9-31.1	0.982
60–69 vs. > 70	0.20	-23.1-23.5	1.000

**Table 30:** Tukey’s test post-hoc analysis for peak SBP categories for women

Comparison	Mean	95% CI	*p*-value
< 40 vs. 40–49	16.16	-9.8-42.1	0.425
< 40 vs. 50–59	16.90	-5.7-39.5	0.240
< 40 vs. 60–69	19.76	-3.4-43.0	0.135
< 40 vs. > 70	26.32	1.6–51.0	< 0.05
40–49 vs. 50–59	0.73	-19.7-21.2	1.000
40–49 vs. 60–69	3.60	-17.6-24.8	0.990
40–49 vs. > 70	10.15	-12.6-32.9	0.732
50–59 vs. 60–69	2.87	-14.0-19.7	0.990
50–59 vs. > 70	9.42	-9.4-28.2	0.639
60–69 vs. > 70	6.55	-13.0-26.1	0.887

**Table 31:** Tukey’s test post-hoc analysis for peak RPP categories for men

Comparison	Mean	95% CI	*p*-value
< 40 vs. 40–49	2940.68	-1048.5-6929.9	0.251
< 40 vs. 50–59	3614.78	-324.7-7554.3	0.088
< 40 vs. 60–69	3375.42	-144.5-6895.3	0.067
< 40 vs. > 70	2063.63	-2185.3-6312.6	0.661
40–49 vs. 50–59	674.10	-3144.7-4493.0	0.988
40–49 vs. 60–69	434.74	-2949.6-3819.1	0.996
40–49 vs. > 70	877.06	-3260.3-5014.4	0.976
50–59 vs. 60–69	239.36	-3086.2-3564.9	1.000
50–59 vs. > 70	1551.16	-2538.3-5640.6	0.829
60–69 vs. > 70	1311.79	-2375.1-4998.7	0.860

**Table 32:** Tukey’s test post-hoc analysis for peak RPP categories for women

Comparison	Mean	95% CI	*p*-value
< 40 vs. 40–49	1423.23	-2366.1-5212.5	0.838
< 40 vs. 50–59	500.85	-2795.5-3797.2	0.993
< 40 vs. 60–69	599.56	-2790.6-3989.7	0.988
< 40 vs. > 70	1367.99	-2234.7-4970.6	0.832
40–49 vs. 50–59	922.37	-2065.2-3910.0	0.913
40–49 vs. 60–69	2022.78	-1068.0-5113.5	0.373
40–49 vs. > 70	2791.22	-531.2-6113.7	0.144
50–59 vs. 60–69	1100.41	-1361.3-3562.1	0.731
50–59 vs. > 70	1868.84	-878.1-4615.8	0.333
60–69 vs. > 70	768.43	-2090.4-3627.3	0.946

**Table 33:** Tukey’s test post-hoc analysis for exercise time categories for men

Comparison	Mean	95% CI	*p*-value
< 40 vs. 40–49	34.24	-97.7-166.2	0.951
< 40 vs. 50–59	101.92	-30.0-233.9	0.209
< 40 vs. 60–69	133.48	17.1-249.9	< 0.05
< 40 vs. > 70	130.75	-6.8-268.3	0.071
40–49 vs. 50–59	67.68	-58.5-193.9	0.572
40–49 vs. 60–69	99.24	-10.5-209.0	0.096
40–49 vs. > 70	96.51	-35.5-228.5	0.259
50–59 vs. 60–69	31.56	-78.2-141.3	0.930
50–59 vs. > 70	28.83	-103.1-160.8	0.974
60–69 vs. > 70	2.73	-113.6-119.1	1.000

**Table 34:** Tukey’s test post-hoc analysis for exercise time categories for women

Comparison	Mean	95% CI	*p*-value
< 40 vs. 40–49	22.44	-119.0-163.9	0.992
< 40 vs. 50–59	62.31	-62.0-186.6	0.639
< 40 vs. 60–69	94.85	-32.5-222.2	0.245
< 40 vs. > 70	153.25	17.4-289.1	< 0.05
40–49 vs. 50–59	39.87	-71.0-150.7	0.858
40–49 vs. 60–69	72.41	-41.9-186.7	0.407
40–49 vs. > 70	130.81	7.1-254.5	0.032
50–59 vs. 60–69	32.54	-59.6-124.7	0.866
50–59 vs. > 70	90.94	-12.6-194.5	0.114
60–69 vs. > 70	58.40	-48.8-165.7	0.562

ANOVA showed significant age-related differences in height (*p* < 0.05), resting SBP (*p* < 0.05), peak HR (*p* < 0.05), peak SBP (*p* < 0.05), and exercise time (*p* < 0.05). Weight (*p* = 0.110), BMI (*p* = 0.399), baseline HR (*p* = 0.944), and peak RPP (*p* = 0.07) were not significantly different across age groups.

Post-hoc analysis indicated that height decreased significantly with advancing age, with differences between < 40 vs. 60–69, 40–49 vs. 60–69, 50–59 vs. 60–69, and 60–69 vs. > 70 (*p* < 0.05). Resting SBP was significantly higher in the 60–69 age group compared with < 40 years (*p* < 0.05). Peak HR decreased significantly across almost all age comparisons, including < 40 vs. 50–59, < 40 vs. 60–69, < 40 vs. > 70, 40–49 vs. 50–59, 40–49 vs. 60–69, 40–49 vs. > 70, 50–59 vs. > 70, and 60–69 vs. > 70 (*p* < 0.05). Peak SBP was significantly higher in older groups compared to < 40 years (< 40 vs. 50–59, < 40 vs. 60–69, < 40 vs. > 70; *p* < 0.05). Exercise time was reduced in men aged 60–69 compared to those aged < 40 years (*p* < 0.05).

### Statistical analysis comparing age categories for women

Table 35 shows ANOVA analysis of baseline and exercise data across age categories in women. Tables 17, 18, 19, 20, 21, 22, 23, 24, 25, 26, 27, 28, 29, 30, 31, 32, 33 and 34 show Tukey’s test post-hoc analysis across age categories for men and women separately.

**Table 35:** Single factor ANOVA comparing age categories for women

Variable	Age groups compared	df	F-statistic	*p*-value
Weight, kg	< 40, 40–49, 50–59, 60–69, > 70	4	2.46	< 0.05
Height, cm	< 40, 40–49, 50–59, 60–69, > 70	4	20.03	< 0.05
BMI, kg/m^2^	< 40, 40–49, 50–59, 60–69, > 70	4	1.27	0.285
Baseline HR, bpm	< 40, 40–49, 50–59, 60–69, > 70	4	1.10	0.361
Resting SBP, mmHg	< 40, 40–49, 50–59, 60–69, > 70	4	6.20	< 0.05
Peak HR, bpm	< 40, 40–49, 50–59, 60–69, > 70	4	62.66	< 0.05
Peak SBP, mmHg	< 40, 40–49, 50–59, 60–69, > 70	4	2.26	0.07
Peak RPP	< 40, 40–49, 50–59, 60–69, > 70	4	1.74	0.145
Exercise Time, s	< 40, 40–49, 50–59, 60–69, > 70	4	3.49	< 0.05

ANOVA showed a statistically significant difference between age groups for weight (*p* < 0.05), height (*p* < 0.05), resting SBP (*p* < 0.05), peak HR (*p* < 0.05), and exercise time (*p* < 0.05). BMI (*p* = 0.285), baseline HR (*p* = 0.361), peak SBP (*p* = 0.07), and peak RPP (0.145) were not significantly different.

Tukey’s post-hoc analysis demonstrated that height significantly differed between multiple age groups: <40 vs. 50–59, < 40 vs. 60–69, 40–49 vs. 50–59, 40–49 vs. 60–69, 50–59 vs. > 70, and 60–69 vs. > 70 (*p* < 0.05). Resting SBP was significantly higher in older age groups, with differences between < 40 vs. 60–69, < 40 vs. > 70, 40–49 vs. > 70, and 50–59 vs. > 70 (*p* < 0.05). Peak HR declined significantly with age, with all pairwise comparisons showing statistical significance (*p* < 0.05). Peak SBP differed significantly between < 40 and > 70 years (*p* < 0.05). Exercise time was significantly reduced in the > 70 age group compared with < 40 years (*p* < 0.05).

## Discussion

This study analysed data from 260 patients with normal SBSE results. There were sex-related differences at baseline across all age cohorts, women had a higher heart rate and lower SBP than men. Although women had a higher peak heart rate and lower peak SBP than men, when accounting for the differences in the baseline metrics for these parameters, the change was similar between sexes. There was a significant difference between exercise performance between men and women, including exercise time and peak RPP.

Age was a significant predictor of SBP and exercise time. Older patients had a similar resting heart rate but a higher baseline systolic blood pressure. At peak, age was associated with higher peak SBP, lower peak heart rate and lower exercise time. Given these differences, the peak RPP remained the same across age groups, as even though the heart rate response was attenuated, the blood pressure component increased, meaning effectively workload was the same. Therefore, from this study it can be concluded SBSE consistently measured a patient’s maximal exercise capacity regardless of age.

In addition, RPP offers prognostic and diagnostic insights that extend beyond conventional measures of exercise capacity. In populations with chronic heart failure, RPP has been shown to carry prognostic value by predicting all-cause mortality and therefore offering clinicians an additional parameter for risk stratification [14]. Notably, patients who achieved higher RPP values during exercise had better survival outcomes, reinforcing its role as a meaningful marker of exercise capacity and prognosis. This study allows clinicians to identify abnormal responses and enhances the prognostic value of RPP data obtained during SBSE.

SBSE has benefits over treadmill stress echocardiography, providing increased sensitivity for the detection of myocardial ischaemia [15]. Compared with treadmill, SBSE is associated with a lower peak heart rate but greater increase in systolic blood pressure [10]. Due to these differences, exercise time using exercise treadmill testing cannot be extrapolated to the supine bicycle.

To date, only normal ranges for SBSE have been determined with the addition of cardiopulmonary exercise testing; Kaminsky et al. [16] acquired data from the FRIEND registry, which included 1717 tests on men and 2777 on women, of age ranges 20 to 79 years. In this study, there was significant differences (*p* < 0.1) between men and women in the measurements of maximal workload (watts) and absolute VO2 max (L/min), with men values being greater by 44% and 41% respectively. In comparison to this study, there was a significant difference between men and women for all physiological measurements recorded (*p* < 0.05), with men having an average maximal workload (Watts) 50.0% greater than women, throughout all age cohorts. As we do not routinely perform cardio-pulmonary exercise testing at the time of SBSE, the results are difficult to apply in this setting.

Exercise-capacity is a well-documented predictor of overall cardiovascular health, and it provides valuable insights into the functional status of the heart. Myers et al. [4] retrospectively analysed 6213 men who underwent treadmill exercise testing for clinical reasons. The patient cohort was then further divided into those with (abnormal test results) and without (normal test results) cardiovascular disease. Exercise capacity was recorded in METs, and mortality was tracked with an average follow-up of 6.2 years. During the follow up, it was found deceased individuals had lower exercise capacity, HR and blood pressure during peak exercise. These findings confirmed that peak exercise capacity is the strongest predictor for mortality, with each 1-MET increase in exercise capacity linked to a 12% reduction in mortality risk.

Exercise capacity during exercise testing is the key prognostic tool for clinicians to utilise, and the data from this study has established normal reference ranges across age groups and sexes, allowing this key prognostic data to be commented on in study conclusions.

### Study limitations

Given the retrospective design of this study, there were study limitations to consider. Firstly, the cohort consisted of patients who were referred for SBSE due to already existing symptoms, indicating uncertainty that these individuals were free from cardiovascular disease. This has the potential to introduce bias, as our reference ranges may not fully represent truly asymptomatic healthy populations. Nonetheless, this does reflect the typical clinical scenario, as SBSE is predominantly performed on symptomatic patients for the purpose of further investigation, thus making our findings relevant for this specific patient population. We chose to include patients with resting hypertension in our study. We felt it important to include these patients in our dataset as (1) this reflects a real-world population and means the results are generally applicable to all patients attending the echo lab for SBSE, (2) excluding hypertensive patients would mean the cohort of studied patients would be significantly reduced and (3) we felt important to mirror the study design of similar studies evaluating reference ranges for cardio-pulmonary exercise testing and exercise treadmill testing [4, 16].

Secondly, this study included relatively small sample sizes at the extreme ends of the age spectrum. This does constrain the applicability of our reference ranges to very young and very old patients, suggesting a need for further studies with larger and more age-diverse cohorts to validate and expand on our original reference values. Despite these potential limitations, this study has contributed valuable data to the field and demonstrates the importance of establishing and refining normal reference ranges for SBSE. Further studies could analyse data from a larger proportion of patients to include more samples at the extremes of ages or potentially recruit asymptomatic individuals who are free from cardiovascular diseases and risk factors. Mortality outcome data on this cohort would also provide additional insights into the relevance of exercise performance in this setting.

A further limitation of this study is the lack of direct measurement of oxygen uptake. While the study considered METs, it remains unclear whether RPP can serve as an index for predicting exercise capacity or prognosis in SBSE in a manner comparable to METs. As oxygen uptake was not assessed, direct comparisons between RPP and METs are not possible.

## Conclusion

This study presents novel data on supine bicycle exercise parameters in patients with normal stress echocardiograms for both men and women, across all ages from < 40 to > 70. Although men and women have differing baseline HR and SBP, change during exercise is similar. With progressive age, there is an attenuation in heart rate response to exercise but an increase in SBP during exercise. As a result, although advancing age is associated with a reduced exercise time, peak RPP is independent of age and could be used as the most accurate assessment of exercise performance.

## Data Availability

All data generated or analysed during this study are included in this published article.

## Abbreviations

SBSE: Supine bicycle stress echocardiography
SBP: Systolic blood pressure
DBP: Diastolic blood pressure
HR: Heart rate
RPP: Rate pressure product
CTCA: Computed tomography coronary angiography
CAD: Coronary artery disease
ECG: Electrocardiogram
PCI: Percutaneous coronary intervention
LV: Left ventricle
RV: Right ventricle
METs: Metabolic equivalents
SD: Standard deviation

## References

[1] Sicari R, Cortigiani L. The clinical use of stress echocardiography in ischemic heart disease. Cardiovasc Ultrasound. 2017;15(1):7. 28327159 10.1186/s12947-017-0099-2 PMC5361820

[2] Bruce RA, Kusumi F, Hosmer D. Maximal oxygen intake and nomographic assessment of functional aerobic impairment in cardiovascular disease. Am Heart J. 1973;85(4):546–62. 4632004 10.1016/0002-8703(73)90502-4

[3] Edvardsen E, Hansen BH, Holme IM, Dyrstad SM, Anderssen SA. Reference values for cardiorespiratory response and fitness on the treadmill in a 20- to 85-year-old population. Chest. 2013;144(1):241–8. 23287878 10.1378/chest.12-1458

[4] Myers J, Prakash M, Froelicher V, Do D, Partington S, Atwood JE. Exercise capacity and mortality among men referred for exercise testing. N Engl J Med. 2002;346(11):793–801. 11893790 10.1056/NEJMoa011858

[5] Wu XP, Li YD, Zhang M, Zhu WW, Cai QZ, Jiang W, et al. Impaired left ventricular mechanics and functional reserve are associated with reduced exercise capacity in patients with hypertrophic cardiomyopathy. Echocardiography. 2019;36(2):266–75. 30600556 10.1111/echo.14241

[6] Sharma V, Newby DE, Stewart RAH, Lee M, Gabriel R, Van Pelt N, et al. Exercise stress echocardiography in patients with valvular heart disease. Echo Res Pract. 2015;2(3):89–98. 26795878 10.1530/ERP-15-0015 PMC4676429

[7] Sicari R, Nihoyannopoulos P, Evangelista A, Kasprzak J, Lancellotti P, Poldermans D, et al. Stress echocardiography expert consensus Statement–Executive summary: European association of echocardiography (EAE) (a registered branch of the ESC). Eur Heart J. 2009;30(3):278–89. 19001473 10.1093/eurheartj/ehn492

[8] Dagianti A, Penco M, Bandiera A, Sgorbini L, Fedele F. Clinical application of exercise stress echocardiography: supine bicycle or treadmill? Am J Cardiol. 1998;81(12a):g62–7. 10.1016/s0002-9149(98)00056-3 9662230

[9] Modesto KM, Rainbird A, Klarich KW, Mahoney DW, Chandrasekaran K, Pellikka PA. Comparison of supine bicycle exercise and treadmill exercise doppler echocardiography in evaluation of patients with coronary artery disease. Am J Cardiol. 2003;91(10):1245–8. 12745112 10.1016/s0002-9149(03)00275-3

[10] Badruddin SM, Ahmad A, Mickelson J, Abukhalil J, Winters WL, Nagueh SF, et al. Supine bicycle versus post-treadmill exercise echocardiography in the detection of myocardial ischemia: a randomized single-blind crossover trial. J Am Coll Cardiol. 1999;33(6):1485–90. 10334412 10.1016/s0735-1097(99)00043-1

[11] Fisman EZ, Frank AG, Ben-Ari E, Kessler G, Pines A, Drory Y, Kellermann JJ. Altered left ventricular volume and ejection fraction responses to supine dynamic exercise in athletes. J Am Coll Cardiol. 1990;15(3):582–8. 2303627 10.1016/0735-1097(90)90630-8

[12] Pellikka PA, Arruda-Olson A, Chaudhry FA, Chen MH, Marshall JE, Porter TR, et al. Guidelines for Performance, Interpretation, and application of stress echocardiography in ischemic heart disease: from the American society of echocardiography. J Am Soc Echocardiogr. 2020;33(1):1–e418. 31740370 10.1016/j.echo.2019.07.001

[13] Wilkins LW. American College of Sports Medicine. ACSM’s resource manual for guidelines for exercise testing and prescription. Philadelphia (PA): Lippincott Williams & Wilkins; 2012.

[14] Chaikijurajai T, Finet JE, Wu Y, Harb SC, Grodin JL, Jaber WA, Tang WHW. Risk stratification with the haemodynamic gain index and peak rate-pressure product in patients with chronic heart failure undergoing treadmill exercise testing. Eur J Prev Cardiol. 2025;32(11):939–46. 39913190 10.1093/eurjpc/zwaf046

[15] Hecht HS, DeBord L, Sotomayor N, Shaw R, Dunlap R, Ryan C. Supine bicycle stress echocardiography: peak exercise imaging is superior to postexercise imaging. J Am Soc Echocardiogr. 1993;6(3 Pt 1):265–71. 8333974 10.1016/s0894-7317(14)80062-x

[16] Kaminsky LA, Imboden MT, Arena R, Myers J. Reference standards for cardiorespiratory the Fitness Registry and the Importance of Exercise National Database (FRIEND) Registry. Mayo Clin Proc. 2017;92(2):228–233. 10.1016/j.mayocp.2016.10.003 27938891

